# G protein-coupled receptors: The choreographers of innate immunity in *Caenorhabditis elegans*

**DOI:** 10.1371/journal.ppat.1009151

**Published:** 2021-01-21

**Authors:** Siddharth R. Venkatesh, Varsha Singh

**Affiliations:** Department of Molecular Reproduction, Development and Genetics, Indian Institute of Science, Bangalore, India; University of Wisconsin Medical School, UNITED STATES

*Caenorhabditis elegans* is a free-living, bacterivorous nematode that frequently encounters pathogens while foraging for food in decomposing vegetation. Like other invertebrates, *C*. *elegans* entirely relies on its innate immune system to combat invading pathogens. A basal flight or fight response of animals is also observed in worms against infection. The former is an aversion response of *C*. *elegans* against select pathogens, and the latter is an inducible innate response comprising of pathogen-specific effectors including lysozymes, lectins, antimicrobial peptides (AMPs), and cytoprotective molecules. Although pathogen recognition in worms is poorly understood, various signaling pathways and immune effectors facilitating defense response are well studied, making this nematode an excellent model to study host–microbe interactions. In higher vertebrates such as mice and humans, sensing of infection through pathogen-associated molecular patterns (PAMPs) or host damage-associated molecular patterns (DAMPs) is primarily mediated by the toll-like receptors (TLRs), nod-like receptors (NLRs), RIG-I like receptors (RLRs), C-type lectin domain (CTLD) proteins, and AIM-like receptors (ALRs) [1]. Humans have 10 TLRs that sense PAMPs and DAMPs. However, TOL-1, the only TLR homolog in *C*. *elegans*, does not seem to be essential during infections with most pathogens, except during *Salmonella enterica* [2] and *Serratia marcescens* [3] infections. The *C*. *elegans* RIG-I like receptor DRH-1 detects products of viral replication and activates an intracellular pathogen response [4]. CLEC-39 and CLEC-49, two CTLD proteins in *C*. *elegans*, are essential for immune responses against *S*. *marcescens* and are known to bind live bacteria [5]. Despite all these findings, the molecular mechanisms involved in pattern recognition by *C*. *elegans* during a majority of infections remain elusive. In this review, we examine the roles of G protein-coupled receptors (GPCRs) as noncanonical pattern recognition receptors (PRRs) and also discuss how GPCR signaling in *C*. *elegans* regulates various immune processes.

GPCRs form the largest superfamily of cell surface receptors in eukaryotes; *C*. *elegans* encodes approximately 1,300 genes encoding putative GPCRs [6]. They are involved in a variety of physiological processes [7] and also for detecting various environmental cues, including bacterial secondary metabolites [8]. In recent years, several studies on infection in *C*. *elegans* have revealed the importance of GPCRs and their signaling in host defense. In this review, we examine the role of GPCRs in innate immunity via the modulation of “flight” or “fight” responses of *C*. *elegans*.

## Neuronal GPCRs as regulators of innate immunity

**Fig 1:**
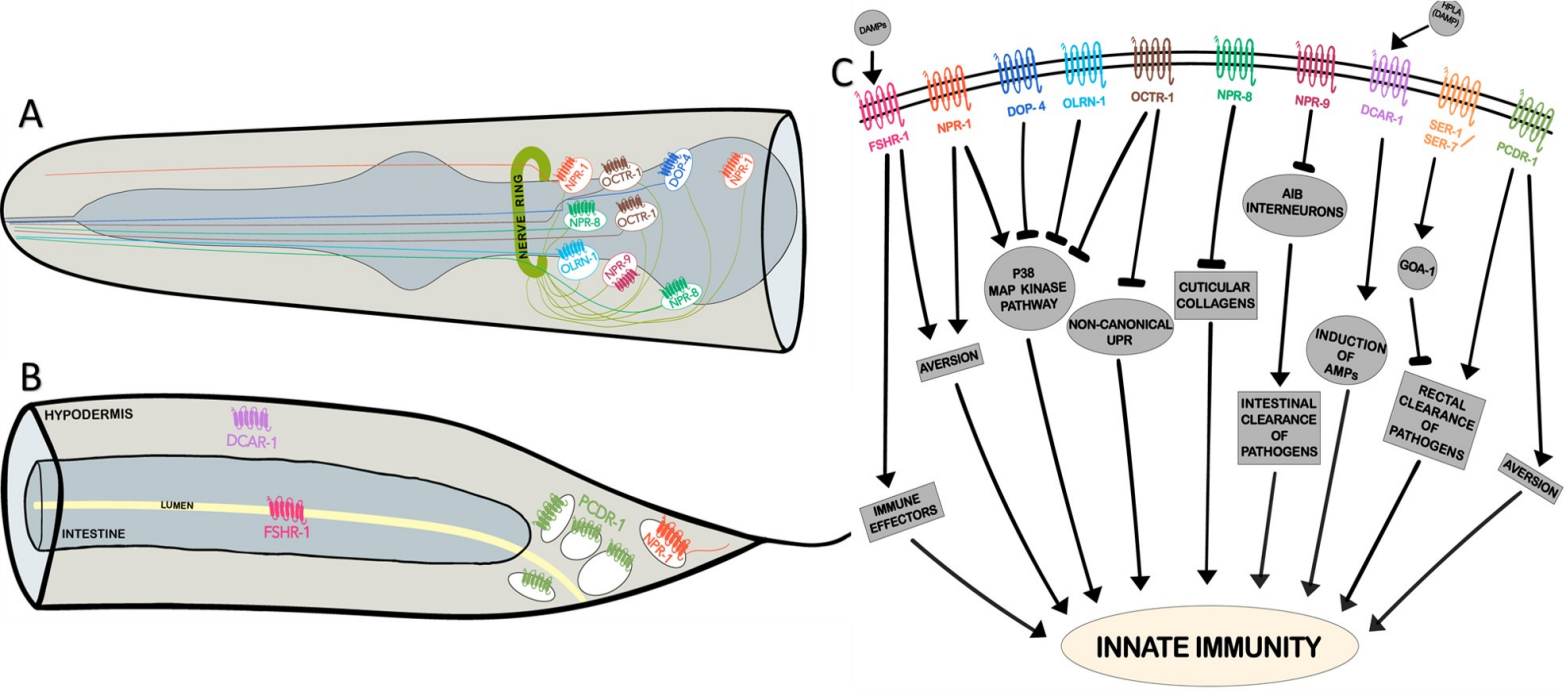
G protein-coupled receptors in *Caenorhabditis elegans* regulate innate immunity in the worm. (A) A lateral view of the head region, representing the neurons expressing the immunomodulatory GPCRs, NPR-1, NPR-8, NPR-9, OLRN-1, DOP-4, and OCTR-1. (B) A lateral view of the posterior end representing the regions/cells expressing the immunomodulatory GPCRs, FSHR-1, DCAR-1, PCDR-1, and NPR-1. (C) A schematic overview of GPCRs regulating various immune response components in *C*. *elegans*. AMP, antimicrobial peptides; DAMP, damage-associated molecular pattern; GPCR, G protein-coupled receptor; HPLA, 4-hydroxyphenyllactic acid; UPR, unfolded protein response.

The nervous system is the primary site of sensory perception and signal integration in animals, making it ideal for systemic regulation of immune responses. The search for PRRs and immune regulators in *C*. *elegans* prompted many research groups on a quest to identify neuronal GPCRs or GPCR signaling components that are involved in immune regulation. A screen of 40 GPCR mutants by Styer and colleagues in 2008 led to the first demonstration of a neuronal GPCR as a regulator of innate immunity in *C*. *elegans* [9]. A mutation in NPR-1, homolog of the human Neuropeptide Y receptor, led to enhanced susceptibility of worms to bacterial infection by *Pseudomonas aeruginosa*, *Enterococcus faecalis*, and *S*. *enterica* [9]. NPR-1 activity in AQR, PQR, and URX sensory neurons was necessary for the activation of p38/MAP kinase pathway, a pro-immunity pathway in *C*. *elegans* (Fig 1A–1C). In contrast, OCTR-1, a catecholamine receptor expressed in a different subset of neurons, was found to regulate innate immunity against *P*. *aeruginosa* negatively [10]. OCTR-1 activity in ASH and ASI neurons suppressed the activation of p38/MAP kinase pathway and a noncanonical unfolded protein response (UPR) pathway (Fig 1A and 1C). The latter consisted of *pqn*/*abu* family of proteins regulated by the phagocyte receptor CED-1 [10]. The β-arrestin ARR-1, a component of GPCR desensitization mechanism in the nervous system, is necessary for the regulation of immune responses to *P*. *aeruginosa*, *S*. *enterica*, and *E*. *faecalis*. Arrestin signaling in ASH and ASI neurons also regulates *pqn/abu* expression during *P*. *aeruginosa* infection [11]. NPR-1 and OCTR-1 provide an example of opposing regulation of p38/MAP kinase pathway by sensory GPCRs.

A recent study illustrated the role for yet another neuronal GPCR in the regulation of innate immunity by modulating intestinal p38/MAPK activity. OLRN-1 is a GPCR required for differentiation of AWC olfactory neurons during larval development. Foster and colleagues showed that OLRN-1 also represses p38/MAPK signaling in the intestine, thereby suppressing unchecked immune activation [12] (Fig 1A and 1C). Another GPCR that suppresses immune responses by inhibiting the p38/MAPK pathway is the D1-like dopamine receptor, DOP-4 [13]. Dopamine secreted from the CEP neurons activates DOP-4 in the ASG neurons. Activation of DOP-4 results in the suppression of the p38/MAPK pathway in a cell-nonautonomous manner (Fig 1A and 1C). Immune regulatory roles of OCTR-1, OLRN-1, and DOP-4 represent homeostatic mechanisms to suppress hyperimmune activation or inflammation. This mechanism appears to be crucial in worms, because the hyperactivation of p38/MAPK hampers *C*. *elegans* growth and development [14], and the hyperactivation of DAF-16 causes inflammation-like death in worms [15]. Two additional neuronal GPCRs also suppress immune activation in worms. Yu and colleagues showed that NPR-9, the human gastrin-releasing peptide receptor ortholog expressed in AIB interneurons (Fig 1A), antagonizes the pro-immune role of AIB interneurons [16]. AIB interneurons prevent the colonization of *C*. *elegans* intestine by *P*. *aeruginosa* (Fig 1C). Meanwhile, NPR-8 GPCR in the AWB, ASJ, and AWC neurons (Fig 1A) is required during infections with *P*. *aeruginosa*, *Staphylococcus aureus*, or *S*. *enterica*. It negatively regulates immunity by suppressing the expression of cuticular collagens such as COL-101, COL-160, and COL-179 involved in the maintenance of cuticle integrity [17] (Fig 1C). All these studies suggest that several neuronal GPCRs are indispensable for survival and immunity in *C*. *elegans* during infection. It is interesting to note that majority of the neuronal GPCRs studied so far repress immune responses.

### Intestinal GPCRs involved in host defense

A unique feature of many PRRs is the presence of a conserved leucine-rich region (LRR) in the ligand-binding domain. Powell and colleagues screened for LRR domain-containing receptors in *C*. *elegans* to identify potential PRRs. They identified FSHR-1 GPCR, ortholog of human follicle stimulating hormone receptor, as a critical regulator of immune response [18]. *fshr-1* mutants show enhanced susceptibility to infection with *P*. *aeruginosa*, *S*. *aureus*, and *E*. *faecalis*. FSHR-1 acts in parallel with the p38/MAPK and regulates a subset of *P*. *aeruginosa* immune-response genes such as *F56D6*.*2*, *C17H12*.*8*, and *F49F1*.*6* [18] (Fig 1C). Using tissue-specific knockdown, the authors demonstrated that FSHR-1 primarily acts in the intestine to regulate survival of worms during infection (Fig 1B). FSHR-1 is also implicated in response to heavy metal and oxidative stress and induces the expression of GCS-2 (an enzyme involved in glutathione biosynthesis) [19]. Further, intestinal FSHR-1 is required for aversion response of worms to *P*. *aeruginosa* [19]. These studies suggest that FSHR-1 may recognize a DAMP released during infection and also during oxidative injury. Thus far, FSHR-1 remains the only intestinal GPCR with an immunomodulatory function.

### Epidermal GPCR regulates defense against fungal invasion

*Drechmeria coniospora* is an ascomycetes fungus and a natural pathogen of *C*. *elegans*. The fungal conidia attach to the worm’s cuticle and germinate, causing the hyphae to penetrate the cuticle, eventually colonizing the entire body of the worm. In response to this invasion, worms up-regulate several AMPs such as neuropeptide-like protein NLP-29 in the epidermis. NLPs in this cluster are also up-regulated during epidermal wounding. The NLP-29 up-regulation during wounding and *D*. *coniospora* infection relies on G_α_ subunit GPA-12 and G_β_ subunit RACK-1 [20] suggesting the involvement of GPCRs. In an RNA interference (RNAi) screen of 1,150 GPCR encoding genes, dihydrocaffeic acid receptor-1 (DCAR-1) was found necessary for NLP-29 up-regulation via p38/MAPK pathway. DCAR-1 responds to the endogenous ligand, 4-hydroxyphenyllactic acid (HPLA), a product of tyrosine degradation in *C*. *elegans* hypodermis. HPLA levels increase during injury and *D*. *coniospora* infection, activating DCAR-1, and subsequently induces immune response in the hypodermis (Fig 1C). Although DCAR-1 is expressed in sensory neurons as well, it is the hypodermal DCAR-1 that responds to injury and infection. Thus, DCAR-1 has a hypodermis-specific function during *D*. *coniospora* infection and wounding [21] (Fig 1B). So far, DCAR-1 remains the only GPCR identified as a DAMP sensor in worms.

### Regulation of immune response by GPCR signaling in the rectal epithelium

Infections with coryneform bacterium *Microbacterium nematophilum*, a natural pathogen of *C*. elegans, results in deformed anal region (Dar). The Dar phenotype can be easily visualized due to the swelling in the rectal epithelium, adjacent to the tail of worms. This is caused by the attachment of bacterial cells to the rectal and post-anal cuticle, and the colonization of the rectal opening, causing swelling, a classic signature of inflammation. During *M*. *nematophilum* infection, Gα_q_ subunit EGL-30 in the rectal epithelium initiates a cascade of signaling events leading to the Dar phenotype [22]. This cascade includes Ras signaling that activates Raf-ERK-MAP kinase signaling cascade, resulting in cell morphology changes in the rectal epithelium. The Dar phenotype promotes pathogen clearance in the rectum and protects the host [23]. Serotonin signaling via SER-1 and SER-7 receptors activate GOA-1, a Gα_o_ subunit, in the rectal epithelium, thereby suppressing the Dar phenotype and immunity [23]. The involvement of GOA-1 and EGL-30 suggested roles for one or more GPCRs in the rectal epithelium. More recently, Anderson and colleagues found an orphan receptor PCDR-1 (pathogen clearance defective receptor-1) to be necessary for the clearance of *M*. *nematophilum* from the rectal opening [24] (Fig 1C). PCDR-1 is expressed in B, F, K, and U rectal cells (Fig 1B). Its activity in these rectal epithelial cells, along with EGL-30, is critical for effective pathogen clearance in the rectum [24]. PCDR-1 also promotes aversion to *M*. *nematophilum* (Fig 1C), but the aversion phenotype does not fully account for the pathogen clearance defects in the mutant [24]. Interestingly, EGL-30 loss-of-function (*lf*) mutation completely abrogates the Dar phenotype, while PCDR-1 *lf* mutation only causes partial suppression of the Dar phenotype [24]. Given this, it is likely that other GPCRs are involved in the regulation of Dar response.

### Chemosensory GPCRs in flight response

*C*. *elegans* has a well-developed chemosensory system that enables it to detect food cues and pheromones. The nematode is repelled by various pathogens, a response termed as “aversion.” It is an example of the flight response, a behavioral strategy conserved in the animal kingdom. Aversion can be categorized into innate and learned aversion. The former is reliant on the worm’s innate ability to detect naturally repulsive components of the pathogen, thereby triggering an innate aversive response. On the other hand, learned aversion involves the process of associative learning upon exposure to pathogens, resulting in the ability to avoid the pathogen on subsequent encounters. Innate aversion is observed when worms are exposed to a lawn of pathogenic bacterium *S*. *marcescens* [3,25]. This response is dependent on the detection of serrawettin W2 (a surfactant secreted by *S*. *marcescens*) [25] and also requires the activity of TOL-1, since *tol-1* mutants are defective in the aversion response toward *S*. *marcescens* [3]. An unidentified chemosensory GPCR in the AWB neurons likely detects Serrawettin W2 [25]. Other examples of flight response-regulating GPCRs are NPR-1 and PCDR-1. Mutations in NPR-1 result in defective “flight” response to *P*. *aeruginosa* [26], while that in PCDR-1 impairs aversion to *M*. *nematophilum* [24] (Fig 1C). Aversion to *P*. *aeruginosa* is of the “learned” nature. Worms are initially attracted to a lawn of *P*. *aeruginosa*, and only begin to avoid the pathogen after a prolonged exposure [10]. Recent studies have shown that this learned aversion is contributed by detection of the metabolites of the pathogens and also by the bloating of the intestine during intestinal infection by *P*. *aeruginosa*. Intestinal infection and the subsequent bloating of the lumen trigger feedback systems that modulate the NPR-1 GPCR pathway that facilitates the regulation of aversive learning [27,28]. The sensors that recognize intestinal bloating are yet to be identified, and it would be interesting to see if FSHR-1 (an intestinal DAMP sensor discussed in a previous section) plays any role in this process.

Interestingly, there are evidences that some pathogens exploit the nematode’s chemosensory system by secreting attractive metabolites to trap them. For example, *S*. *marcescens* secretes 2-butanol and acetone that initially attracts worms, perhaps facilitating an opportunity for the bacteria to establish infection [29]. Similarly, 2-heptanone secreted by *Bacillus nematocida* B16 attracts nematodes via the neuronal GPCR STR-2 [30]. Remarkably, STR-2 GPCR also regulates longevity, through its control of lipid metabolism in the intestine [31]. Since stored fats can boost immune responses [32], it remains to be investigated if STR-2 can regulate survival of worms on *B*. *nematocida* and other Bacillus species. The identification of microbial ligands inducing behavioral response in *C*. *elegans* is an area of active investigations in many labs with a promise to elucidate a GPCR-based model of pattern recognition in the animal kingdom.

### Perspective

In this review, we examined a number of elegant studies in *C*. *elegans* demonstrating immune-modulatory roles of neuronal and nonneuronal GPCRs. Notably, some studies in drosophila, mice, and humans [7] also suggest the involvement of GPCRs in immune responses although not in the same level of detail as in worms. The future holds great promise with the possibility that abundant bacterial metabolites such as ketones, alcohols, esters, sensed by *C*. *elegans* sensory neurons, serve as PAMPs in *C*. *elegans* and other animals. Worms provide us with a unique opportunity to identify additional GPCRs and their ligands with the view of devising pharmacological strategies for boosting innate immune responses and for suppressing inflammation.
